# A Preliminary Research Study for Distribution Characteristics and Sources of Indoor Air Pollutants in the Valuable Archive of the National Library of Korea

**DOI:** 10.3390/ijerph18041715

**Published:** 2021-02-10

**Authors:** Hye-Won Lee, Jeong-In Jeon, Hui-Been Lim, Kwi-Bok Lee, So-Yeon Park, Cheol-Min Lee

**Affiliations:** 1 Department of Health Science, Korea University, Seoul 02841, Korea; gpdnsis@korea.ac.kr; 2 Institute of Risk Assessment, SeoKyeong University, Seoul 02713, Korea; 3 Department of Nano & Biological Engineering, SeoKyeong University, Seoul 02713, Korea; hhzz01@skuniv.ac.kr (J.-I.J.); hibin016@skuniv.ac.kr (H.-B.L.); 4 Research Center of the Records Preservation, National Library of Korea, Seoul 06579, Korea; kuibok@korea.kr (K.-B.L.); vet16@korea.kr (S.-Y.P.)

**Keywords:** indoor air quality, library, valuable archive, national archives, VOCs, aldehydes, pollutant sources, principal component analysis

## Abstract

Important records can be damaged directly and indirectly. Their restoration, if possible, is difficult as it is very time-consuming and costly. Although measures have been taken to permanently preserve records, most studies focus on preventing short-term damage from physical or biological factors and not on preventive measures against chemical damage from long-term polluted air exposure. This study investigated the types, concentrations, and distribution characteristics of hazardous chemicals present in the valuable archive of the National Library of Korea (NLK) and identified the sources of these pollutants. Mean SO_2_, NO_X_, CO, CO_2_, and total volatile organic compound (TVOC) concentrations were 1.49 ± 0.44 ppb, 30.52 ± 19.70 ppb, 0.75 ± 0.21 ppm, 368.91 ± 32.23 ppm, and 320.03 ± 44.20 µg/m^3^, respectively, meeting the Ministry of the Interior and Safety (MOIS) of Korea standards. Toluene (66.43 ± 10.69 µg/m^3^) and acetaldehyde (157.23 ± 6.43 µg/m^3^) were present at the highest concentrations, respectively. Two principal components were extracted via a principal component analysis; the primary component (66%) was closely related to outdoor pollution sources and the secondary component (33%) to indoor sources. Results contribute to establishing air quality standards and management measures for preservation of this archive.

## 1. Introduction

From the perspective of archival science, records (which can be produced by individuals, corporations, or government agencies during their daily business) include books, documents, maps, photographs, or other forms of documentation that are created or obtained in relation to the original business activities in accordance with legal obligations, regardless of their physical form and characteristics. Records are preserved by an agency or its legitimate successor as evidence of its functions, policies, decisions, and procedures, or due to the informational value of the data they contain [1]. These records not only are a means for information transmission, but also have very important historical and cultural values, so it is necessary to establish and apply appropriate preservation measures for each record [2]. 

Damage to such records can be categorized into direct and indirect effects. Direct effects include disasters, such as earthquakes, while damage from indirect effects is caused by long-term exposure to environmental factors, such as the record storage conditions [3,4]. Once damaged, it is difficult to restore such records and, if possible, restoration requires significant time and cost, so damage to records due to negligence in management is a great financial loss and damages our cultural heritage, as they provide testimony of national history. Hence, many countries are devoting effort to developing various preservation technologies for libraries, storage, and museums to preserve their history [5], and an efficient system to manage and preserve records is being actively researched in part of standardizing the preservation environment and facilities [6].

Air pollutants are known to affect not only human health but also the permanence of substances [7,8,9,10]. In particular, sulfur dioxide (SO_2_), nitrogen oxides (NO_x_), and ozone (O_3_) are major pollutants from the outdoors that cause damage to heritage. These pollutants are of great concern in the preservation facilities, so indoor air monitoring is routinely performed [11,12]. As a result of efforts to reduce air pollutants over the past several years, however, the concentration of these pollutants in the outdoor air and the indoor air of preservation facilities decreases. In recent years, the focus of indoor air quality (IAQ) is shifting from outdoor pollutants such as SO_2_, NO_2_, and O_3_ to indoor pollutants such as organic acids [13,14]. Currently, most of the measures for permanent preservation of records are to prevent damage caused by physical and biological factors that can cause damage in a short period of time, the measures to prevent chemical damage due to prolonged exposure to contaminated air have not been established [15]. 

Organic acids and aldehydes such as acetaldehyde and formaldehyde are known to mainly exist in the preservation environment of paper records [9,16,17,18], and separately from human activities and outdoor air inflow, the decomposition of paper and other cellulosic materials is known to be the main source of volatile organic compounds (VOCs) in the library [19,20,21,22,23,24,25]. VOCs such as organic acids and aldehydes must be managed in terms of long-term preservation of records, as they can cause accelerated deterioration of paper; accordingly, the Ministry of the Interior and Safety (MOIS) of Korea has established air quality standards for preservation environments, including formaldehyde (HCHO), total volatile organic compounds (TVOC), etc. [26]. However, the standards follow the Korean Atmospheric Environment and Indoor Air Quality Standard, set by the Ministry of Environment (MOE), which is unsuitable for the preservation and management of records [27].

This work was conducted as a preliminary study to prepare measures for establishing air quality standards for preservation environments in paper records archives to investigate the types, concentration, and distribution of hazardous chemical pollutants that are present in the valuable records archive of the National Library of Korea (NLK) and identify the source of these hazardous chemical pollutants to provide a reference for establishing air quality standards and management measures for the preservation environment of repositories.

## 2. Materials and Methods 

### 2.1. Investigation Subject and Sampling Point

This study focused on the valuable archive, which houses paper records in the NLK. The building, located in Seocho-gu, Seoul, was completed in August 2000 and has a collection area of 588 m^2^. The structure is divided into two floors; old and rare books are stored on the first floor, and general rare books are stored on the second floor. The majority of the bookshelves were constructed from glued and laminated pinewood timber, the floor was constructed from beech, and the ceiling and walls were constructed with humidity control panels. Therefore, the interior of the valuable archive contains a large amount of wood. An air conditioner was implemented to control the temperature, humidity, and indoor air quality. This study was conducted during summer, when air is supplied from 07:30 to 08:30 at a flow rate of 18,900 m^3^/h and recirculated from 09:00 to 17:30 at a flow rate of 2000 m^3^/h. The valuable archive is disinfected twice a year using natural anti-microbial products and pesticides. The roof of the valuable library is partially waterproofed on the cement, and a flower bed is formed on one side. One of the other three sides is adjacent to the road, and two are adjacent to the parking lot.

Indoor and outdoor VOCs were collected from the valuable archive on 1 July 2019, at 10 am, 1 pm, and 6 pm, a total of 3 times, and other gas substances including SO_2_, NO, NO_2_, CO, and CO_2_ were measured for 24 h, 1–2 July 2019. Sampling was conducted at locations that represented the air quality of the whole room, i.e., near the centers of the first and second floors and roof of the building.

### 2.2. Measurement and Analysis Method

The hazardous chemical pollutants measured in this study included SO_2_, NO, NO_2_, CO, CO_2_, total volatile organic compounds (TVOC; i.e., the pollutants to be controlled in general indoor and repository environments designated by the MOE and MOIS of Korea), 21 VOCs, and 11 aldehyde pollutants that are likely to appear in the repository environment. 

The SO_2_, NO, NO_2_, CO, and CO_2_ levels were measured following the Korean Atmospheric Environment and Indoor Air Quality Standard Method set by the MOE. The concentrations of all substances (excluding TVOCs) were investigated for 24 h to include the periods before and after operation of the air conditioner and every hour to determine the characteristics of the valuable archive, including the effect of air conditioner operation. Table 1 lists the devices and methods used to measure SO_2_, NO_X_, CO, and CO_2_ concentrations.

The concentrations of TVOCs and 21 VOCs (i.e., hexane, dichloromethane, chloroform, 1,2-dichloroethane, benzene, trichloroethane, toluene, tetrachloroethane, ethylbenzene, 1,3-butadiene, 4-methyl-2-pentanone, m-xylene, p-xylene, o-xylene, styrene, carbon tetrachloride, hexadecane, vinyl chloride, butyl acetate, isobutyl alcohol, and methyl-ethyl-ketone) were measured and analyzed following the Korea Standard Method for Indoor Air Quality Test of the MOE [28]. VOCs samples were collected using a mini-pump and Tenax-TA solid adsorbent tube 1 m from the floor at a flow rate of 0.1 L/min for 30 min. After collecting samples, both ends of the adsorbent tube were sealed, and the tube was refrigerated at ≤4 °C. The adsorbent tubes containing samples were analyzed by a gas chromatography/mass spectrometry detector (GC/MSD) after thermal desorption was analyzed using a thermal desorption device. The GC/MSD and thermal desorption (TD) conditions employed when analyzing the VOCs are summarized in Table 2. The limit of detection (LoD) was ≤10 ng, and the values less than LoD are expressed as N.D. 

The concentrations of 11 aldehydes were measured following the Korea Standard Method for Indoor Air Quality Test of the MOE. The aldehydes were collected using a mini-pump and DNPH (2,4-dinitrophenylhydrazine) cartridge, to which an ozone scrubber was attached to prevent the influence of ozone when collecting samples. Samples were collected 1 m from the floor at a flow rate of 0.1 L/min for 30 min, and both ends of the cartridge were sealed with plastic caps before refrigerating the cartridge at ≤4 °C. The DNPH derivative of the DNPH cartridges containing samples was extracted using an acetonitrile solvent, and a portion of the solution was quantified using a 360 nm UV detector through high-performance liquid chromatography (HPLC). The HPLC conditions for the analysis of aldehydes are summarized in Table 3. LoD was ≤20 ng, and the values less than LoD are expressed as N.D.

### 2.3. Pollutant Source Identification 

To identify the pollutant sources, multivariable analyses, such as principal component analysis (PCA), were conducted to determine the effects of various pollutants on the indoor air quality of the repository. PCA is a widely used statistical method in environmental epidemiology and is a receptor methodology that analyzes the physical and chemical characteristics of pollutants; identifies the sources of pollutants affecting air, water, and soils; and quantifies the degree of influence for the effective management of environmental pollution [29].

In this study, PCA was conducted to identify the sources of pollutants present in the valuable archive. First, to resolve the lack of reliability of measured data, 240 possible concentrations with a normal probability distribution were generated based on the mean and standard deviation of the concentration of each pollutant, which was used for data analysis. Data were generated using the R package and analyzed using IBM SPSS Statistics 18 for PCA.

## 3. Results

### 3.1. Distribution of Standard Pollutants

The SO_2_, NO_X_ (NO, NO_2_), CO, CO_2_, and TVOC contents in the valuable archive, first and second floor of the building, and outside area, are summarized in Table 4. The concentrations on the first and second floors were averaged and taken as the mean indoor concentrations. Additionally, the following standards were considered to compare the degree of pollution caused by each pollutant: Air Quality Standards in the Preservation Environment, specified in the Public Records Management Act by the Ministry of the Interior and Safety of Korea; Air Quality Standards in Public Facilities set by the Indoor Air Quality Management Act by the MOE; and ISO-11799 international standards.

The average indoor SO_2_ and NO_X_ (NO + NO_2_) concentrations in the preservation environment were 1.49 ± 0.44 and 30.52 ± 19.70 ppb, respectively, which met the standards set by the MOIS; however, the NO_X_ concentration was approximately three to six times higher than the ISO-11799 standards. Therefore, the current NO_X_ standards must be reviewed for more effective management. Furthermore, the indoor/outdoor (I/O) ratio of the concentrations of these two pollutants was > 1, indicating that the indoor concentration exceeded the outdoor concentration. 

The mean indoor CO, CO_2_, and TVOCs concentrations in the preservation environment were 0.75 ± 0.21 ppm, 368.91 ± 32.23 ppm, and 320.03 ± 44.20 µg/m^3^, respectively, satisfying the standards set by the MOE, as was the case for SO_2_ and NO_X_. The I/O concentration ratio of CO to CO_2_ was <1, indicating that the outdoor concentration was higher than the indoor concentration in the repository. In contrast, the ratio for the TVOCs was >1, indicating that the indoor concentration exceeded the outdoor concentration, as was the case for SO_2_ and NO_X_.

### 3.2. VOC Distribution 

Table 5 lists the concentration distributions of VOCs detected in the indoor and outdoor environments of the valuable archive; the concentration distributions of only six (toluene, hexane, methyl-ethyl-ketone, benzene, isobutyl alcohol, ethylbenzene) of the 21 surveyed VOCs exceeded the LoD. Among the VOCs, ethylbenzene was not detected indoors, suggesting that it is not released by the environment of the valuable archive. Five VOCs were identified in the valuable archive, i.e., toluene, hexane, methyl-ethyl-ketone, benzene, and isobutyl alcohol, and the mean concentrations of these five VOCs in the indoor air of the valuable archive were 66.43 ± 10.67, 16.27 ± 1.91, 12.27 ± 1.21, 6.43 ± 0.23, and 4.00 µg/m^3^, respectively.

These findings show that future measures to manage VOCs in the indoor air of valuables archive should focus on these six detected VOCs.

### 3.3. Aldehyde Distribution 

Table 6 summarizes the concentration distributions of aldehydes in the indoor and outdoor air of the repository environment, and the concentration distributions of only nine (acetaldehyde, formaldehyde, propionaldehyde, butylaldehyde, hexanal, pentanal, crotonaldehyde, benzaldehyde, i-valeraldehyde) of the eleven surveyed aldehydes exceeded the LoD; their mean concentrations in the indoor air of the repository were 157.23 ± 6.43, 26.87 ± 1.79, 6.03 ± 1.03, 5.10 ± 0.96, 3.00 ± 1.05, 2.09 ± 0.61, 2.53 ± 1.54, 2.25 ± 0.49, and 1.10 µg/m^3^, respectively.

The I/O concentration ratio was <1 for hexanal, crotonaldehyde, and i-valeraldehyde, suggesting that the outdoor area was the major source of these pollutants and was >1 for acetaldehyde, formaldehyde, propionaldehyde, butylaldehyde, pentanal, and benzaldehyde.

### 3.4. Principal Component Analysis

In this study, we conducted PCA, a multivariate analysis method, to estimate the sources of the hazardous chemical pollutants in the valuable archive, and the results are summarized in Table 7.

**Figure 1:**
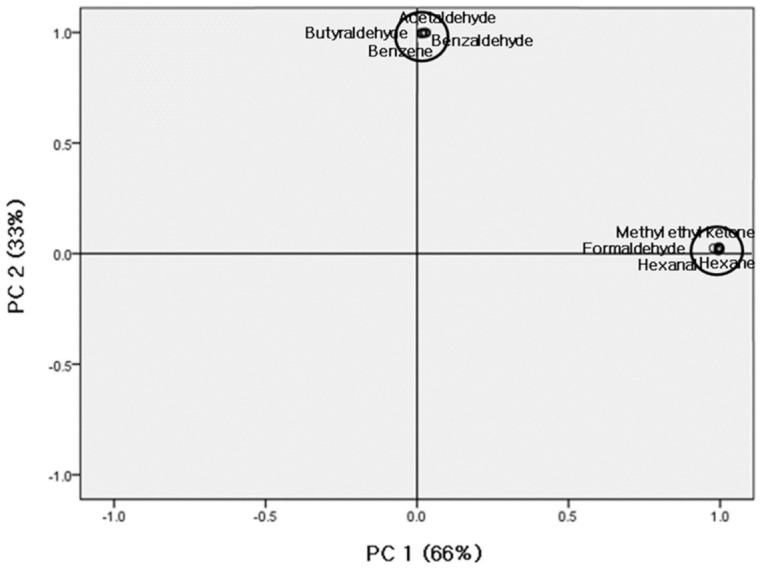
Principal component analysis (PCA) of data in the valuable archive environment.

The 18 target pollutants were analyzed, and two principal components were extracted (Figure 1). The first and second principal components accounted for 66.0% and 33.0% of the total, respectively.

The first principal components were highly correlated with formaldehyde, hexanal, hexane, methyl ethyl ketone, propionaldehyde, toluene, CO, CO_2_, NO, NO_2_, SO_2_, and TVOCs. To identify the common source of the first principal component (PC1), information regarding the known sources of these pollutants is summarized in Table 8. The pollutants extracted as the PC1 were substances with outdoor sources, including automobile parts, automobile fuel, and air pollution. Therefore, they were considered to have been introduced by the outdoor air when the air conditioner operated in supply.

The second principal component (PC2) included acetaldehyde, benzaldehyde, benzene, butylaldehyde, crotonaldehyde, and pentanal, and their concentrations were found to be highly correlated with one other. To identify the common sources of these pollutants, information regarding their sources is summarized in Table 9. The pollutants extracted as the PC2 were found to have indoor sources, including paper, prints, construction materials, wood, and glue. These pollutants are considered to have been released by the old books and wooden bookshelves used to store the old books in the valuable archive.

## 4. Discussion

Various physical, chemical, and biological factors are involved in damaging records and cultural properties [5,30,31,32,33]; however, research on chemical factors is more limited than that on physical and biological factors and the preparation of management measures. Therefore, this work was conducted as a pilot study to prepare measures to define the causes of damage to records and cultural properties and to prepare management measures by identifying the types, concentrations, and distribution characteristics of hazardous chemical pollutants that can be found in records preservation environments to provide a reference for establishing standards for air quality and management measures for the preservation environment.

As mentioned above, this was a preliminary study conducted over a short time period in the preservation environment of the valuable archive; therefore, the obtained data are very limited. To overcome this limitation, the concentrations of pollutants detected through several measurements collected in a short time period were used with statistical techniques to calculate and utilize the concentrations that might appear. Additionally, numerous investigations have been conducted into the hazardous indoor air pollutants of the reading rooms or exhibition halls of general libraries and museums in Korea [5,27,34,35,36,37]. However, almost no researchers have focused on hazardous chemicals in the indoor air of preservation environments, with limited access to the personnel of valuable archives. Therefore, this study, which was the first to investigate the types, concentration, and distribution characteristics of the hazardous chemical pollutants present in valuable archives, is expected to be valuable as a basic reference for future research.

The findings of this study show that the concentrations of SO_2_, NO_X_ (NO + NO_2_), CO, CO_2_, and TVOC (i.e., the hazardous chemical pollutants defined by the air quality standards for preservation environments by the Public Records Management and Indoor Air Quality Management Acts of the MOE) did not exceed the current limits. However, the standards were not created by conducting systematic investigation to prevent damage to records in preservation environments; rather, they were based on the Korean Atmospheric Environment and Indoor Air Quality Standard Method set by the MOE, which aims to prevent harmful effects to humans and is insufficient for the preservation and management of records in valuable archives.

The results were compared to the ISO-11799 standards for SO_2_ and NO_X_; the SO_2_ concentration met the standards; however, the NO_X_ concentration exceeded them by three to six orders of magnitude. According to previous research, NO_X_ is a hazardous environmental pollutant contributing to the deterioration of paper records, along with temperature and humidity, even in preservation environments [38,39]. Based on this result, an effective NO_X_ management system for valuable archives must be developed. The I/O ratio for hazardous chemical pollutants, along with the established standards, indicated that the indoor concentration exceeded the outdoor concentration of some pollutants, indicating that they originated from indoor sources. However, the PCA results exhibited the opposite result, indicating the existence of pollutants with outdoor sources. This finding can be interpreted as follows: These pollutants are mostly substances emitted during combustion; however, there are no combustion facilities in or around the valuable archive, indicating that there is no indoor source of emissions. However, both indoor and outdoor NO concentrations increased around 7 to 9 o’clock in the early morning, which was when traffic was heavy and when the supply air was provided. Therefore, considering the result of the average indoor NO concentration according to whether or not the air conditioner is operated, the NO concentration is considered to be affected by the operation of the air conditioner (supply air). In the case of NO_2_, it is thought that it is affected by outdoor air like NO. That is, it is believed that NO flowed in the archive by the supplied air, and this NO was converted into NO_2_ by a chemical reaction in the air.

The valuable archive, which was the target of this study, has almost no floating population or indoor activities, and the operation of an air conditioner without an exhaust function aggravated the difficulty in ventilation. Therefore, a higher indoor concentration of some pollutants than their outdoor concentration may have been due to the accumulation of pollutants that entered from the outside and were trapped indoors. There are no management standards for the concentrations of VOCs and aldehyde pollutants, domestically or internationally, and it could not be determined whether the concentration in the valuable archive was sufficient to cause the deterioration of the records in the preservation environment. Nevertheless, given the results of previous studies on the effect of these pollutants on the deterioration of preserved records, deterioration and damage evaluation should be conducted [9,22,23,38,40] to prepare standards for pollutants. The indoor concentrations of only 5 of the 21 surveyed VOCs and 9 of the 11 aldehyde pollutants were found to exceed the LoD. This suggests that the air of different repositories may contain different types of hazardous chemical pollutants, which demonstrates the importance of considering the characteristics of each repository when formulating tailored measures to manage hazardous chemical pollutants in preservation environments. Additionally, the indoor concentrations of most VOCs and aldehyde pollutants exceeded the outdoor concentrations, and the PCA results indicated that they originated from indoor sources. This confirms that reducing pollutants in indoor air should receive more focus than controlling the inflow of outside air when establishing measures to manage such pollutants.

## 5. Conclusions

This work was conducted as a pilot study to prepare measures to develop air quality standards for the preservation environments of paper records repositories by identifying the types, concentrations, and distribution characteristics of hazardous chemical pollutants in the valuable archive of the NLK and defining their sources. The results of this study can be summarized as follows:

1. Concentrations of domestically and internationally designated substances for management were determined, and the mean concentrations of SO_2_, NO_X_ (NO + NO_2_), CO, CO_2,_ and TVOCs were 1.47 ± 0.11 ppb, 5.56 ± 4.06 ppb, 23.67 ± 2.70 ppb, 0.72 ± 0.09 ppm, 367.47 ± 27.28 ppm, and 320.03 ± 44.20 µg/m^3^, respectively, which meet the standards for public records management set by the MOIS of Korea; however, the TVOCs and NO_X_ concentrations approached the acceptable limits set by the domestic and international ISO-11799 standards.
2. Twenty-one target VOCs were measured, and the following six substances were detected: hexane, benzene, toluene, ethylbenzene, isobutyl alcohol, and methyl-ethyl-benzene; ethylbenzene was not detected indoors. Of the five VOCs detected indoors, the toluene concentration was highest, at 66.43 ± 10.69 µg/m^3^, followed by hexane, methyl-ethyl-ketone, benzene, and isobutyl alcohol. The I/O ratios were > 1 for toluene, hexane, and benzene, indicating that these pollutants originated from indoor sources.
3. Only 9 of the 11 analyzed aldehydes were detected, including formaldehyde, acetaldehyde, propionaldehyde, crotonaldehyde, butylaldehyde, benzaldehyde, i-valeraldehyde, pentanal, and hexanal, and concentrations of the other aldehydes were below the LoD. The indoor acetaldehyde concentration was the highest (157.23 ± 6.43 µg/m^3^), followed by formaldehyde, propionaldehyde, butylaldehyde, hexanal, pentanal, crotonaldehyde, benzaldehyde, and i-valeraldehyde.
4. According to the PCA results, two principal components were extracted. PC1 and PC2 accounted for 66.0% and 33.0% of the total, respectively. PC1 contained formaldehyde, hexanal, hexane, methyl-ethyl-ketone, propionaldehyde, toluene, CO, CO_2_, NO, NO_2_, SO_2_, and TVOCs, which were correlated with outdoor sources. PC2 contained acetaldehyde, benzaldehyde, benzene, butylaldehyde, crotonaldehyde, and pentanal, which were found to share indoor sources.

Based on these findings, we could indirectly identify problems associated with the domestic standards for records management that need to be improved. As well as the existing substances considered in management, high concentrations of individual VOCs, aldehydes, and organic acids were detected, and additional measures are required to manage these substances. Despite the potential limitations in the measurement results due to the short research period, this study is significant as we estimated the sources of hazardous chemical pollutants through PCA and is the first study on the types and concentration distribution characteristics of hazardous chemical pollutants in preservation environments with limited public access excluding personnel, such as valuable archives.

## Figures and Tables

**Table 1:** SO_2_, NO_2_, CO, and CO_2_ measurement methods and instruments.

Pollutants	Measurement and Analysis Method	Measurement Instruments
SO_2_	UV Fluorescence	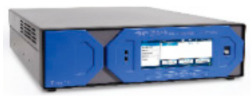
NO_x_	Chemi-luminescent Detection	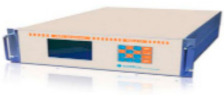
CO	Non-Dispersive Infrared absorption	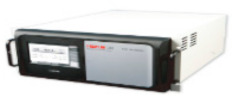
CO_2_	Non-Dispersive Infrared absorption	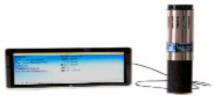

**Table 2:** Gas chromatography (GC), mass spectrometry (MS), and thermal desorption (TD) analyses conditions.

TD Conditions	
Desorption temperature	300 °C (relative with sorbent)
Desorption flow	30 mL/min, 15 min
Focusing trap	Liquid N_2_ or sorbent
Desorption of focusing trap	325 °C
**GC Conditions**	
Injector temperature	300 °C
Carrier gas	He, 1.8 mL/min
Column	OV-1 capillary column (0.32 mm × 60 m × 1 µm)
Temperature program	50 °C (50 min)–8 °C /min–200 °C (until all target compounds elute)
**MS Conditions**	
Interface temperature	250 °C
Ion source temperature	200 °C
Ionization	Electron ionization (70 eV)
Mass range	Scan, m/z 35 to m/z 350

**Table 3:** High-performance liquid chromatography (HPLC) analysis conditions.

Parameter	Conditions
Column	C-18 column (Octadecyl-silica (ODS), 250 mm × 4.6 mm)
Mobile phase	Acetonitrile/Water = 60/40 (*v*/*v*)
Detector	360 nm UV
Carrier flow rate	1.0 mL/min
Sample injection volume	20 µL

**Table 4:** Concentrations of air pollutants in the valuable archive and air pollution standards. TVOC—total volatile organic compounds.

Pollutant	Standard		Mean ± S.D.				I/O Ratio
	MOIS * (Korea)	ISO-11799 ^†^ (International)	Indoor			Outdoor	
			1st Floor	2nd Floor	Total		
SO_2_ (ppb)	50	5–10	1.88 ± 0.13	1.10 ± 0.25	1.49 ± 0.44	1.12 ± 0.61	1.33
NO (ppb)	50(NO_x_)	5–10(NO_2_)	5.99 ± 8.34	5.29 ± 4.29	6.72 ± 6.71	3.65 ± 4.34	1.56
NO_2_ (ppb)			11.25 ± 3.08	36.11 ± 3.81	23.80 ± 12.99	4.71 ± 2.14	5.18
CO (ppm)	10	-	0.85 ± 0.19	0.57 ± 0.11	0.75 ± 0.21	1.58 ± 0.19	0.46
CO_2_ (ppm)	1000 ^‡^	-	360 ± 21.71	373 ± 36.96	368.91 ± 32.23	422 ± 3.75	0.88
TVOC (µg/m^3^)	400	-	320.03 ± 44.20	-	320.03 ± 44.20	15.53 ± 4.78	21.96

* Ministry of the Interior and Safety, Korea ^†^ ISO-11977—Document storage requirements for archives and library materials ^‡^ Ministry of Environment, Korea.

**Table 5:** Volatile organic compounds (VOCs) concentrations in the valuable archive. I/O ratio—indoor/outdoor ratio.

Pollutant	Mean ± S.D. (µg/m^3^)		I/O Ratio
	Indoor	Outdoor	
Toluene	66.43 ± 10.69	7.87 ± 3.36	8.44
Hexane	16.27 ± 1.91	9.60 ± 2.12	1.69
Methyl ethyl ketone	12.27 ± 1.21	-	-
Benzene	6.43 ± 0.23	4.20 ± 0.71	1.53
Isobutyl alcohol	4.00	-	-

**Table 6:** Aldehyde concentrations in the valuable archive. I/O ratio—indoor/outdoor ratio.

Pollutant	Mean ± S.D. (µg/m^3^)		I/O Ratio
	Indoor	Outdoor	
Acetaldehyde	157.23 ± 6.43	11.33 ± 11.49	13.87
Formaldehyde	26.87 ± 1.79	16.23 ± 15.43	1.66
Propionaldehyde	6.03 ± 1.03	2.15 ± 0.35	2.81
Butylaldehyde	5.10 ± 0.96	2.50 ± 0.14	2.04
Hexanal	3.00 ± 1.05	3.60	0.83
Pentanal	2.90 ± 0.61	1.40	2.07
Crotonaldehyde	2.53 ± 1.54	4.00	0.63
Benzaldehyde	2.25 ± 0.49	2.25 ± 0.07	1.00
i-Valeraldehyde	1.10	2.40	0.46

**Table 7:** Principal component (PC) analysis results. TVOC—total volatile organic compounds.

Variable	PC1	PC2
Acetaldehyde	0.081	0.992
Benzaldehyde	0.094	0.994
Benzene	0.084	0.995
Butylaldehyde	0.092	0.993
Crotonaldehyde	0.086	0.994
Pentanal	0.097	0.994
Formaldehyde	0.993	−0.038
Hexanal	0.993	−0.045
Hexane	0.997	−0.041
Methyl ethyl ketone	0.995	−0.045
Propionaldehyde	0.996	−0.048
Toluene	0.997	−0.045
CO	0.991	−0.055
CO_2_	0.997	−0.043
NO	0.977	−0.042
NO_2_	0.995	−0.044
SO_2_	0.996	−0.040
TVOC	0.998	−0.047
Eigenvalue	11.900	5.946
% Dispersion	65.965	33.182

**Table 8:** Sources of pollutants in the first principal component (PC1). TVOC—total volatile organic compounds.

Pollutant	Sources
Formaldehyde	Cigarettes, car care products, building materials, fuel, etc.
Hexanal	Cigarettes, air cleaner, etc.
Hexane	Automotive component, tire, automotive fuel, lubricant, traffic, etc.
Methyl Ethyl Ketone	Automotive component, automotive fuel, lubricant, paint, softner, etc.
Propionaldehyde	Cigarettes, rubber, disinfectant, etc.
Toluene	Air cleaner, cigarettes, automotive fuel, building materials, etc.
CO	Automotive, combustion apparatus, air pollution, cigarettes, etc.
CO_2_	Human body, combustion apparatus, etc.
NO	Cigarettes, etc.
NO_2_	Automotive, combustion apparatus, air pollution, cigarettes, etc.
SO_2_	Combustion apparatus, air pollution, etc.
TVOC	-
Common	Cigarettes, automotive, air pollution (outdoor pollutants)

**Table 9:** Sources of pollutants in the second principal component (PC2).

Pollutant	Sources
Acetaldehyde	Paper, adhesive, binding, paint, building material, cleaner, printing, ink, etc.
Benzaldehyde	Adhesive, absorbent, binding, building material, colorant, paint, pesticide, printing, etc.
Benzene	Adhesive, binding, building construction, building material, cleaner, colorant, printing, ink, wood, paper, etc.
Butylaldehyde	Adhesive, building material, paint, printing, cleaner, etc.
Crotonaldehyde	Pesticide, building material, etc.
Pentanal	Disinfectant, rubber, etc.
Common	Adhesive, building material, paint, cleaner, etc. (indoor pollutants)

## Data Availability

The data presented in this study are available on request from the corresponding author.
